# Prediction of Microbial Growth Rate versus Biomass Yield by a Metabolic Network with Kinetic Parameters

**DOI:** 10.1371/journal.pcbi.1002575

**Published:** 2012-07-05

**Authors:** Roi Adadi, Benjamin Volkmer, Ron Milo, Matthias Heinemann, Tomer Shlomi

**Affiliations:** 1 Department of Computer Science, Technion, Haifa, Israel; 2 Institute of Molecular Systems Biology, ETH Zurich, Zurich, Switzerland; 3 Departments of Plant Sciences, Weizmann Institute of Science, Rehovot, Israel; 4 Molecular Systems Biology, Groningen Biomolecular Sciences and Biotechnology Institute, University of Groningen, AG Groningen, The Netherlands; Institute for Systems Biology, United States of America

## Abstract

Identifying the factors that determine microbial growth rate under various environmental and genetic conditions is a major challenge of systems biology. While current genome-scale metabolic modeling approaches enable us to successfully predict a variety of metabolic phenotypes, including maximal biomass yield, the prediction of actual growth rate is a long standing goal. This gap stems from strictly relying on data regarding reaction stoichiometry and directionality, without accounting for enzyme kinetic considerations. Here we present a novel metabolic network-based approach, MetabOlic Modeling with ENzyme kineTics (MOMENT), which predicts metabolic flux rate and growth rate by utilizing prior data on enzyme turnover rates and enzyme molecular weights, without requiring measurements of nutrient uptake rates. The method is based on an identified design principle of metabolism in which enzymes catalyzing high flux reactions across different media tend to be more efficient in terms of having higher turnover numbers. Extending upon previous attempts to utilize kinetic data in genome-scale metabolic modeling, our approach takes into account the requirement for specific enzyme concentrations for catalyzing predicted metabolic flux rates, considering isozymes, protein complexes, and multi-functional enzymes. MOMENT is shown to significantly improve the prediction accuracy of various metabolic phenotypes in *E. coli*, including intracellular flux rates and changes in gene expression levels under different growth rates. Most importantly, MOMENT is shown to predict growth rates of *E. coli* under a diverse set of media that are correlated with experimental measurements, markedly improving upon existing state-of-the art stoichiometric modeling approaches. These results support the view that a physiological bound on cellular enzyme concentrations is a key factor that determines microbial growth rate.

## Author Summary

While current genome-scale metabolic modeling approaches enable us to successfully predict a variety of metabolic phenotypes, identifying the factors that determine microbial growth rate and the prediction of growth rates under various conditions is still an open challenge. Here we present a metabolic network-based approach, MetabOlic Modeling with ENzyme kineTics (MOMENT), which predicts growth rates by integrating standard stoichiometric modeling with prior data on enzyme turnover rates and enzyme molecular weights, considering a physiological bound on total enzymes' concentration. The method is based on a finding that enzymes catalyzing high flux reactions tend to be more efficient in terms of having higher turnover numbers. MOMENT predicts growth rates of *E. coli* across a set of 24 different media that are significantly correlated with experimental measurements, while existing state-of-the art stoichiometric modeling approaches fail to do so. These results suggest that a bound on cellular enzyme concentrations is a key factor that determines microbial growth rate.

## Introduction

Traditional metabolic modeling techniques involve the reconstruction of kinetic models based on detailed knowledge on enzyme kinetic parameters for all enzymes in a certain system [1]. These models are limited to small-scale systems due to lack of sufficient data on kinetic constants and the highly complex nature of these models. An alternative approach called Constraint-Based Modeling (CBM) predicts certain steady-state cellular metabolic phenotypes in microorganisms on a genome-scale by relying solely on simple physical-chemical constraints, without requiring enzyme kinetic data [2], [3], [4]. This approach identifies steady-state flux rates (in units of mmol/(g[DW]*h) through a metabolic network, satisfying stoichiometric mass-balance as well as reaction directionality constraints, such that nutrients taken up with a certain measured rate (in units of mmol/g[DW]*h) are transformed into biomass. The metabolic network includes a biomass production reaction that consumes essential biomass metabolites, with its stoichiometric coefficients representing the molar quantities required for generating a unit mass of cells (in units of mmol/g[DW]). This reaction's flux activity represents the growth rate (in units of 1/h). CBM is now commonly used for metabolic engineering in microorganisms, predicting the effect of gene knockouts on organism viability [2].

Flux Balance Analysis (FBA) is a commonly used CBM approach that enables to predict biomass production yield (in units of gram biomass/gram nutrient) based solely on reactions' stoichiometry and directionality (i.e. without measurements of nutrient uptake rates). Given information only on reactions' stoichiometry and directionality, the prediction of biomass yield works by searching for a feasible flux distribution with maximal flux through the biomass production reaction, considering an arbitrary upper bound on the uptake rate of the carbon nutrient. The maximal biomass production rate predicted by FBA reflects optimal yield metabolism and is equal to the assumed uptake rate multiplied by maximal biomass yield. The prediction of actual growth rate by FBA is theoretically possible when experimental measurement of nutrient uptake rates is available and is used to constrain the uptake flux in the model (or alternatively, by multiplying FBA-predicted biomass yield with the measured uptake rates). However, experimental studies have shown that microorganisms exhibit non optimal-yield metabolism under various conditions, for example, in the case of over-flow metabolism where excess nutrient uptake is metabolized inefficiently [5], [6]. In fact, growth rate was found to be inversely correlated with biomass yield in some microorganisms under different growth environments (see Section 4 in Supp. Material of [7]). Hence, growth rate prediction obtained by FBA (reflecting optimal yield metabolism) are likely to be unrealistically high in many cases. Predicting the correct growth rate even when nutrient uptake rates are known is a challenging task. A more ambitious conceptual challenge is the prediction of growth rate without measurements of nutrient uptake rates under a variety of environmental and genetic conditions.

FBA with Molecular Crowding (FBAwMC) is a recently developed extension of FBA which was shown to enable the prediction of growth rates of *E.coli* across a small set of growth media (without given measurements of nutrients uptake rates), as well as under conditions of over-flow metabolism [8], [9]. This was achieved by accounting for the enzyme concentrations required for catalyzed metabolic flux (utilizing data on enzyme kinetic constants), considering a physiological upper bound on the total cellular volume used by metabolic enzymes. Other recent modeling approaches aim to predict cellular metabolism by integrating molecular crowding constraint with kinetic parameters: (i) A recent study has utilized a variant FBAwMC to predict inefficient metabolism in cancer cells, in accordance with the Warburg effect [10]. (ii) A method by Zhuang et al [11] accounts for a constraint relating to the competition for membrane space between nutrient transporters and respiratory chain proteins was shown to improve metabolic prediction, without requiring explicit data on nutrient uptake rates. (iii) Goelzer et al [12] models cellular metabolism by accounting for both solvent capacity constraints and translation apparatus. Another method recently shown to utilize enzyme turnover numbers to improve metabolic flux prediction is Integrative Omics Metabolic Analysis (IOMA), requiring further quantitative proteomic and metabolomics data as input [13]. Another method that aims to predict cellular metabolism without requiring nutrient uptake rates is E-flux [14], which relies on high-throughput gene expression data (shown to predict growth rates in a qualitative manner). Still, none of these approaches were shown to successfully predict in a quantitative manner the growth rate of microbes across conditions, without utilizing a-priori data on nutrient uptake rates.

In this paper, we present a method, MetabOlic Modeling with ENzyme kineTics (MOMENT), for predicting metabolic fluxes and growth rates by accounting for the maximal cellular capacity for metabolic enzymes without the requirement of experimentally determined uptake rates. Extending upon FBAwMC, MOMENT accurately quantifies the enzyme concentrations required for catalyzing each metabolic reaction based on known kinetic constants, accounting for isozymes, protein complexes and multi-functional enzymes. MOMENT is shown to predict growth rates for *E.coli* under a diverse set of growth media that are significantly correlated with experimental measuements, without requiring measured nutrient uptake rates, significantly outperforming the prediction accuracy of FBAwMC. Furthermore, MOMENT is shown to markedly improve the prediction performance of various metabolic phenotypes, including metabolic fluxes and expression level of metabolic genes. We begin our analysis by exploring the relation between enzyme kinetic parameters and measured metabolic flux, showing a design principle in which enzymes catalyzing high flux reactions across different media tend to be more efficient in terms of having higher turnover numbers (hence requiring lower concentration to achieve a certain flux rate). This suggests that a a physiological constraint on total cellular enzyme concentration, which underlies MOMENT, significantly affects cellular metabolism and the evolution of enzyme kinetic parameters.

## Results

### The evolution of enzyme kinetic parameters optimizes metabolic flux

An enzyme turnover number is defined as the maximal number of molecules of substrate that the enzyme can convert to product per catalytic site per unit of time. We extracted enzyme turnover numbers for 251 reactions from BRENDA [15] and SABIO-RK [16] databases. To infer genome-scale metabolic flux rates, we utilized several dozen metabolic fluxes under various growth rates in glucose minimal media (obtained from Ishii et al. [17] and Schuetz et al. [18]), and integrated them with a genome-scale metabolic network model of *E.coli* [19] to infer the most likely rates through the entire network. Specifically, this was done based on standard quadratic programming optimization by minimizing the Euclidian distance between the predicted and the measured fluxes to fit the predicted fluxes to measured ones [20]. Notably, this analysis does not make usage of kinetic data as input. As an alternative approach for inferring global flux distributions, we employed Flux Balance Analysis, followed by Flux Variability Analysis [21], to identify metabolic reactions whose flux can be uniquely determined based on stoichiometric mass-balance constraints and maximal biomass yield assumption (obtaining overall similar results in the analysis described below for the flux distributions obtained by the two approaches; Table S1; Figure S1).

**Figure 1:**
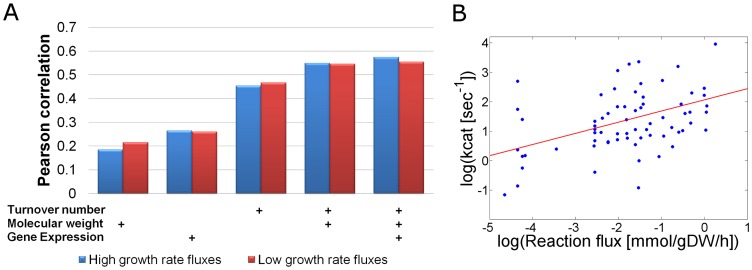
Enzyme turnover numbers and enzyme molecular weights are significantly correlated with metabolic flux rates. (A) Correlations of enzyme turnover numbers, enzyme molecular weights, gene expression levels, and combinations of the latter via a linear regression model with measured metabolic flux rates. Measured flux rates in *E.coli* under glucose minimal media in low and high growth rates were taken from Ishii et al. [17], and Schuetz et al. data [18], respectively. Each bar represents a correlation between flux rates and a single or multiple data sources (marked by ‘+’ signs). (B) Enzyme turnover numbers correlate with measured metabolic flux rates in *E. coli* (both in log_10_ scale). Linear regression line in red.

When comparing the enzyme kinetic parameters in *E. coli* and measured flux rates, we found that enzymes catalyzing high flux reactions have high turnover number, with statistically significant Pearson correlations of 0.45 (*p*-value = 7.8e-5) and 0.46 (*p*-value = 3.6e-5) between turnover rates and fluxes under conditions of low and high growth rates, respectively (Figure 1; considering base 10 log of both fluxes and turnover numbers). These correlations suggest that higher selection pressure for enzymatic efficiency (i.e. higher turnover rates) acts on enzymes carrying high flux reactions. Notably, our results extend upon a recent finding that central metabolic enzymes have higher turnover rates than secondary metabolic enzymes [22], by considering actual flux rates instead of relying on rough categorization of enzymes to primary and secondary metabolism.

Enzyme molecular weights were computed based on genomic sequences, extracted from KEGG [23]. A statistically significant Pearson correlation of 0.22 (*p*-value = 3.2e-5) was also found between metabolic flux rates and enzyme molecular weights, indicating higher flux rates for enzymes with high molecular weights (Figure 1A). Interestingly, a simple linear regression model that aims to explain metabolic flux rates based on both enzyme turnover numbers and molecular weights provided a Pearson correlation of 0.55 (*p*-value = 8.1e-7) with metabolic flux rates, suggesting that each parameter contributes independently to explaining flux rates (Figure 1A).

While enzyme kinetic parameters are scarcely used in genome-scale metabolic modeling approaches, gene expression data is commonly utilized as the basis for metabolic flux prediction [24], [25]. However, computing the correlation between the above described flux rates and gene expression [26] measured also under glucose minimal media, resulted in Pearson correlations of only 0.26 and 0.265 under low and high growth rates, respectively. The latter correlations are markedly lower than those obtained between flux rates and enzyme turnover numbers. This is a remarkable result considering that both the gene expression and metabolic fluxes were measured under the very same growth media, while the kinetic parameters are constant characteristics of the enzymes across different growth conditions. Adding the gene expression data to the above described regression model provided an insignificant contribution to metabolic flux predictions (Figure 1A). Futher utilizing proteomic data for 67 enzyme-coding genes in *E. coli* measured under the same growth media [26], we did not find a significant correlation between protein concentrations and the metabolic flux rates. These findings further highlight the importance of utilizing enzyme kinetic data as a prime data source for metabolic flux prediction.

**Figure 2:**
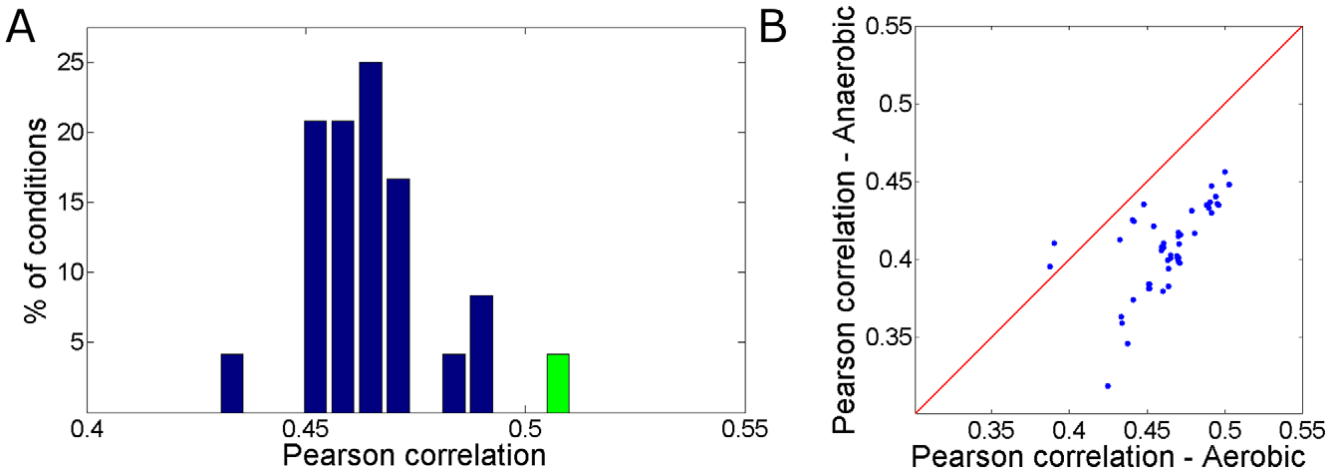
Enzyme turnover rates show higher correlation with average flux across media and with flux under aerobic conditions. (A) Histogram of Pearson correlations between enzyme turnover numbers and predicted flux rates under different single carbon and energy source media (in blue). The Pearson correlation between enzyme turnover numbers and the averaged flux distribution across conditions (in green) is shown to be markedly higher than those obtained under the different media. (B) Pearson correlations between enzyme turnover numbers and predicted fluxes under a set of single carbon and energy source media under either aerobic versus anaerobic conditions. As shown, under most growth conditions, the correlation between enzyme turnover numbers and fluxes is higher when fluxes are predicted under aerobic conditions.

Having shown that enzyme turnover numbers are significantly correlated with measured flux rates under glucose minimal media, we set to examine the correlation between enzyme turnover numbers and flux rates under a diverse set of growth media. Towards this end, we applied FBA to predict likely flux distributions under a set of media (listed in the Methods), setting the growth rate to experimental measurements and optimizing for maximal yield. We find that the average Pearson correlation between the enzyme turnover numbers and the predicted fluxes across these media is 0.46 (Figure 2A). Next, we computed the correlation between the mean flux rate per enzyme across the growth media and enzyme turnover numbers, finding a Pearson correlation of 0.52, which is higher than the correlations obtained under any single medium. This result suggests that enzyme turnover rates may potentially evolve to support efficient metabolism across multiple media. To explore whether metabolism is better tuned for a specific growth medium, we compared the correlation between predicted fluxes and enzyme turnover numbers achieved for aerobic versus anaerobic conditions (Figure 2B). We found that the correlation between predicted fluxes and enzyme turnover numbers is significantly higher in aerobic conditions (paired Wilcoxon test *p*-value = 3e-15), suggesting a potentially stronger selection pressure for efficient metabolism under aerobic conditions. These results suggest that data on enzyme kinetics and metabolic flux may provide valuable insight into organisms' natural environment, in line with previous attempts to do so via other molecular data sources such as codon usage and gene expression [27].

### Utilizing enzyme kinetic parameters within genome-scale metabolic modeling

The fact that enzyme turnover numbers and the molecular weights of enzymes are significantly correlated with metabolic flux rates suggests that the utilization of the latter data sources within metabolic modeling approaches may provide improved prediction accuracy of metabolic phenotypes (as also shown in [9], [10], [11], [12]). Towards this end, we developed a method called MetabOlic Modeling with ENzyme kineTics (MOMENT), which utilizes the kinetic parameters under the limitation of the total enzymatic pool available. Given a growth condition of interest, MOMENT predicts a flux distribution that satisfies stoichiometric mass-balance and reaction directionality constraints, such that the total mass of enzymes required to catalyze the predicted flux is bounded by the total enzymatic mass, considering a similar constraint to that used by FBAwMC and by [12], [21] (Methods). Enzyme turnover numbers are used to compute an upper bound on enzyme concentrations required to catalyze the corresponding flux rates, and enzyme molecular weights to transform concentrations to units of mass. However, unlike FBAwMC, MOMENT jointly searches for a feasible flux distribution and for the corresponding enzyme concentrations required, considering isozymes, enzymatic complexes, and multi-functional enzymes. This is achieved by making usage of detailed gene-to-reaction mapping that is commonly represented in CBM models via Boolean equations (Methods). For isozymes, the gene-to-reaction mapping denotes that the expression of one of several genes is required to catalyze a certain reaction, while for enzyme complexes, that the expression of several genes is jointly required. Notably, the entire set of gene-to-reaction mapping is formulated as part of the linear programming in a recursive manner, without requiring a more complex optimization such as mixed-integer linear programming that is commonly used to model this mapping [28], [29]. For reactions in *E. coli* for which no enzyme turnover numbers were extracted from the above described databases, mean turnover numbers from other species were considered, yielding a total set of turnover numbers for 513 enzymes. Reactions for which no turnover number was available in any species were assigned with the median turnover number across all reactions in *E.coli*, as in [10]. Using only enzyme turnover numbers measured for *E.coli* provided lower prediction accuracy for MOMENT as well as for the other computational approaches, still showing a marked advantage in prediction accuracy to MOMENT (Table S2). An implementation of MOMENT is available via http://www.cs.technion.ac.il/~tomersh/tools/.

### Predicting *E. coli's* growth rate across growth media

**Figure 3:**
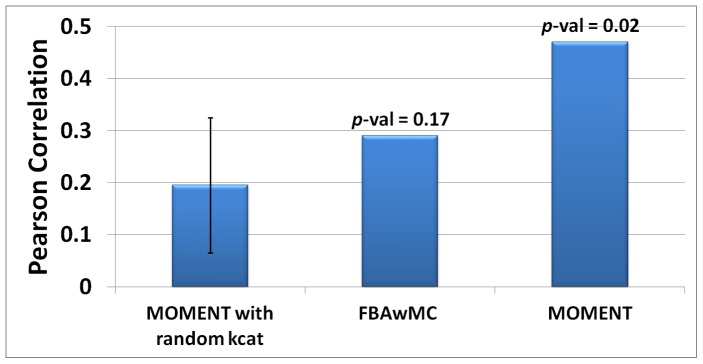
Growth rate prediction accuracy by MOMENT versus other approaches. The prediction of *E.coli* growth rate under 24 different minimal media based on MOMENT, FBAwMC, and MOMENT with random enzyme turnover rates (with the error bar representing standard deviation over 1000 randomly shuffled turnover numbers). As shown, only MOMENT (with the true turnover rates) achieves a statistically significant Pearson correlation between predicted and measured growth rates (*p*-values are shown on top of each bar).

**Figure 4:**
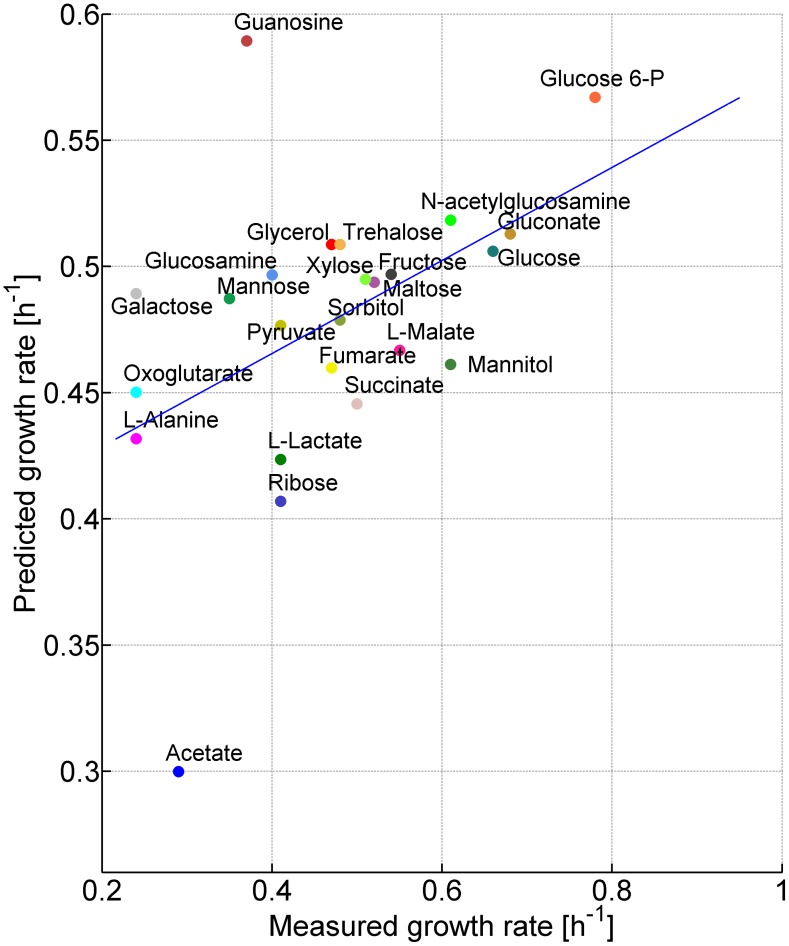
The prediction of growth rates by MOMENT. MOMENT predicted growth rates achieving a Pearson correlation of 0.47 (p-value = 0.02) with the measured growth rates.

To evaluate MOMENT's ability to predict microbial growth rates, we experimentally measured *E. coli's* growth rates on 24 single carbon and energy source media (Methods, Table S3) and compared the predicted and measured rates. The predictions were obtained by applying MOMENT on the genome-scale metabolic network model of *E. coli* iAF1260 [19]. We found that growth rate predictions obtained by MOMENT were significantly correlated with the measured ones, with a Pearson correlation of 0.468 (*p-*value = 0.02; Figure 3 and 4), and a Spearman correlation of 0.473 (p-value = 0.0196). Notably, varying the threshold on the total enzyme mass linearly scales the predicted growth rates (and hence, by definition, does not change the above correlations between predicted and measured growth rates; Text S1).

Protein mass was previously shown to account for 56%(g enzymes/g_DW_) of cellular mass (based on experimental measurements [30]). Assuming that the entire protein mass is allocated to metabolic enzymes, we initially predicted a mean growth rate of 1.02 (1/h) across growth media, which is markedly higher than the mean measured growth rate of 0.47 (1/h). Searching for a threshold on the total enzymatic mass that minimizes the deviation between measured and predicted growth rates (in terms of square differences) resulted in a threshold of 27% (g enzymes/g_DW_) (suggesting that only 48% of protein mass is taken by metabolic enzymes). Hence, practically, in order to scale the predicted growth rates to the correct range, prior knowledge on the total mass of metabolic enzymes should be used. In this case, the identified fraction of proteins mass devoted to metabolic enzymes in *E. coli* is further supported by analyzing gene expression data [26], which show that the sum of expression level of enzyme-coding genes is 35% of the total sum of expression level of all genes.

While the growth rate predictions obtained by MOMENT are significantly correlated with the measured ones, the standard deviation of the predicted rates (across the different media) is markedly lower than that of the measured growth rates (0.054 for predicted rates versus 0.14 for measured rates). A potential explanation for the differences between the standard deviations in the observed and predicted growth rates could be that the fraction of protein mass devoted to metabolic enzymes increases under high growth rates. Notably, checking this hypothesis would require high-throughput protein concentration data measured under various growth rates, though this kind of data is currently unavailable. Overall, utilizing additional experimental data on the total enzyme concentration in a specific growth condition of interest is expected to further improve MOMENT's predictive performance.

To evaluate the importance of the utilized enzyme turnover numbers, we repeated MOMENT's growth rate predictions with randomly shuffled turnover numbers, which were found to provide significantly lower prediction accuracy (Figure 3; *p*-value = 0.026, representing the fraction of random samplings, which have led to a higher correlation with the measured growth rates than that achieved with the known turnover numbers).

To benchmark our new method, we tested the prediction performance of the previously developed FBAwMC. Here, FBAwMC was provided with the very same enzyme turnover rates given to MOMENT, while performing a sampling procedure for missing parameters as described in Beg et al did not improve the predicted performance (data not shown). We found that the growth rate prediction achieved by FBAwMC are not significantly correlated with the measured ones (Figure 3; Figure S2; *p*-value = 0.17), although a significant correlation between measured and predicted growth rates was reported for a smaller set of 10 media by Beg et al. [8]. Notably, the scope of the 24 media considered here is significantly wider as it includes also nucleotides and amino-acids which were not considered in the set of 10 media studied by Beg et al. When focusing on the growth rate measurements for the limited set of 10 media made by Beg et al, both FBAwMC and MOMENT achieve significant Pearson correlations, though insignificant Spearman correlations (see Table S4). A previous study by Wong et al. [31] suggests that growth rate is proportional to the square root of growth yield. We find that MOMENT's predictions satisfy this relation, with a significant correlation (Pearson R = 0.3953, p-value = 0.05; Spearman R = 0.5046, p-value = 0.01) between the predicted growth rate and the square root of the predicted biomass yield.

### Predicting metabolic flux, gene and enzyme expression levels

**Figure 5:**
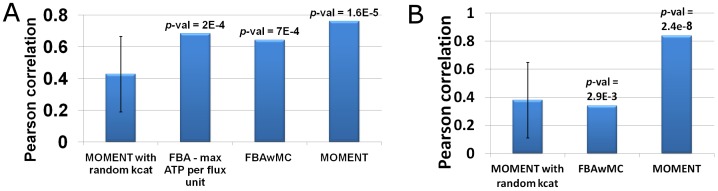
Metabolic flux and gene expression level predictions via MOMENT versus other approaches. (A) The prediction of flux rates in *E.coli* under glucose minimal media based on MOMENT in comparison to FBAwMC, FBA, and MOMENT with random enzyme turnover rates (error bar representing standard deviation over randomly shuffled turnover numbers). (B) The prediction of differential gene expression levels in *E.coli* under glucose minimal media, between low and high growth rate conditions (with the high growth rate condition involving overflow metabolism). *p* values are shown above each bar.

To evaluate the performance of MOMENT in predicting intracellular fluxes, we compared experimental flux measurements for 28 reactions in *E.coli* measured under exponential growth phase by Schuetz et al. [18] with the predicted fluxes. We found that flux predictions obtained by MOMENT achieve a Pearson correlation of 0.76 with the measured fluxes, significantly outperforming FBAwMC and FBA, which achieve correlations of 0.64 and 0.51, respectively. As a further control, we tested a variant of FBA, which maximizes ATP yield per sum of flux square, previously shown by Schuetz et al. to improve flux prediction accuracy [18]. We found that predictions obtained by the latter approach achieve a Pearson correlation of 0.68 with the measured fluxes, which is still markedly lower than MOMENT's prediction accuracy (Figure 5A; Figure S3A; Table S5). Also here, the utilization of the randomly sampled enzyme turnover numbers led to worse predictions (Figure 5A; Table S5).

To further evaluate the predictive performance of MOMENT, we extracted data from [32] on gene expression changes in *E.coli* under glucose minimal media, between low and high growth rate conditions, the latter involving over-flow metabolism, and compared it to predicted changes in enzyme concentrations. Applying MOMENT to predict changes in protein concentrations between these low and high growth rate conditions, we predicted 28 enzymes with a significant change in concentration (deviating from the expected increase in enzyme levels due to the fold change increase in growth rate). We found that changes in these enzyme concentrations predicted by MOMENT between the low and high growth rates achieve a Pearson correlation of 0.84 (*p*-value = 2.4e-8) with the measured changes in gene expression (Figure 5B; Figure S3B; Table S6), even though gene and protein expression levels tend to be only moderately correlated [33]. Also here, the performance of FBAwMC is significantly lower in this case (Figure 5B; Table S6), with a Pearson correlation of 0.34 (*p*-value = 2.9e-3).

Notably, naïve FBA was not evaluated here as it cannot be applied to predict differential metabolism across different growth rates. As a further benchmark, we applied a recently developed method called Parsimonious enzyme usage FBA (pFBA), to classify genes in *E. coli* according to whether they are used in the optimal growth solutions (as this classification was previously shown to correlate with changes in gene expression following laboratory-evolved *E.coli* straints that increased their growth rates) [34]. We found only a weak correlation between this gene classification and the changes in gene expresion between the low and high growth rates conditions [32] (Pearson R = 0.092, p-value = 0.02; Spearman R = 0.074, *p*-value = 0.06).

## Discussion

Computational prediction of microbial growth rates represents a major challenge. Here, we present a novel computational approach, MOMENT, that addresses this challenge by integrating genome-scale metabolic modeling with enzyme kinetic parameters. MOMENT is shown to predict growth rates for *E. coli* under various growth media that are significantly correlatred with experimental measurements, and to improve the prediction accuracy of several metabolic phenotypes including intracellular fluxes, and gene expression of enzyme-coding genes. The method is based on an identified design principle of metabolism, in which enzymes catalyzing high flux reactions across different media tend to have higher turnover numbers.

While MOMENT enables genome-scale prediction of metabolic phenotypes it is bound to make simplifying assumptions that in some cases may lead to false predictions: (i) MOMENT requires as input information on the fraction of total protein concentrations that is devoted to metabolic enzymes. Since this information is difficult to obtain for each modeled condition, here we assumed that this fraction remains constant across a variety of growth media, which is expected to bias the predictions. (ii) MOMENT does not take into account several important factors that affect growth rate such as the cost of protein synthesis by ribosomes and local substrate turnover numbers, etc [35]. (iii) MOMENT requires data on enzyme kinetic constants, which is still unavailable for hundreds of enzymes in *E. coli*. Specifically, kinetic data on various membrane transporters is missing from both BRENDA [15] and SABIO-RK [16], which may lead to false prediction regarding the cost of activating specific transporters and regarding the effect of knocking out transporters. (iv) MOMENT does not predict metabolite concentrations and hence does not take into account thermodynamic considerations (regarding flux directionality) or enzyme saturation considerations in computing required enzyme levels (implicitly assuming that the majority of enzymes in *E. coli* are fully saturated, following [36]). Future studies may extend MOMENT to also predict metabolite concentrations, satisfying the 2^nd^ law of thermodynamics as done in [37], while considering enzyme saturation effects via known enzyme binding affinity constants (Km).

From an applicative standpoint, the improved metabolic modeling performance achieved by MOMENT is expected to significantly contribute to metabolic engineering applications and specifically to optimal strain design. Specifically, the additional constraints employed by MOMENT on the requirement for specific enzyme concentrations for catalyzing predicted metabolic flux rates, can be integrated and potentially improve the accuracy of computational metabolic engineering methods such as OptKnock, RobustKnock, OptStrain, etc [38], [39], [40]. MOMENT's ability to correctly predict microbial growth rates supports the underlying assumption that a physiological bound on cellular enzyme mass is a key factor that determines growth rate.

## Methods

### Growth rate determination

#### Strains and medium

The wild-type Escherichia coli K-12 strain BW25113 was used for all experiments. Cells were cultivated in M9-minimal medium with different carbon source added. The carbon sources were added so that the amount of reducible carbon equaled the amount present in a concentration of 2 g/L glucose. M9 minimal medium was prepared in the following way: To 700 mL of autoclaved, purified water, 200 mL of 5× base salt solution (211 mM Na_2_HPO_4_, 110 mM KH_2_PO_4_, 42.8 mM NaCl, 56.7 mM (NH_4_)_2_SO_4_, autoclaved), 10 mL of trace elements (0.63 mM ZnSO_4_, 0.7 mM CuCl_2_, 0.71 mM MnSO_4_, 0.76 mM CoCl_2_, autoclaved), 1 mL 0.1 M CaCl_2_ solution (autoclaved), 1 mL 1 M MgSO_4_ solution (autoclaved), 2 mL of 500× thiamine solution (1.4 mM, filter sterilized) and 0.6 mL 0.1 M FeCl_3_ solution (filter sterilized) were added. The resulting solution was filled up to 1 liter with water. Carbon source were added to the medium from stock solutions adjusted to pH 7. All media were filtrated prior to use (Steritop-GP 500 mL, Millipore). All chemicals used were obtained from Sigma-Aldrich if not indicated otherwise.

#### Cultivation

The growth rates were determined using an automated cultivation device (Tecan Infinite 200 Pro plate reader). Cells were grown to a steady state in the respective growth medium in shake flasks, then washed twice in minimal medium. 4 µL of the washed cells were inoculated into a well on a 96-well plate (black, Greiner) with 196 µL of medium. The plate was covered with a transparent plastic cover and sealed with parafilm. Cultivation was done at the maximal linear shaking speed (160 min-1, 1 mm displacement) and the following settings for optical density (OD) measurement (Interval time 5 min, shaking 4∶42, reading (no shaking) 18 s; number of flashes 1; wavelength 600 nm, bandwidth 9 nm). The cells were grown to stationary phase or for at least 50 hours to ensure observation of steady state growth. The measured OD-values were corrected for the non-linearity of the device using an empirical function derived from samples with known OD-values (measured by spectrometry) from 10 to 0.001.

### Extraction of enzyme kinetic parameters

Enzyme turnover rates were extracted from BRENDA [15] and SABIO-RK [16]. based on Enzyme Commission (EC) numbers and reactant names in the *E.coli* metabolic model by Feist et al. [19] (Table S7). Measured turnover rates for mutated enzymes were filtered out. When multiple turnover numbers were available for a certain enzyme, the median value was chosen.

### MetabOlic Modeling with ENzyme kineTics (MOMENT)

Similar to FBA, MOMENT searches for a feasible flux distribution vector *v* (mmol/g_DW_/h) with maximal growth rate (i.e. flux through the biomass production reaction), satisfying mass-balance and reaction directionality constraints based on the following linear constraints:

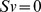
,

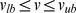
,

Where *S* denotes a stiochiometric matrix *S* (*NxM*) composed of *N* metabolites and *M* reactions (*S_ij_* corresponds to the stoichiometric coefficient of metabolite *i* in reaction *j*) and *v_lb_* and *v_ub_* represent known lower and upper bounds, respectively, on flux rates. Here, *v_lb_* is set to either −inf for reversible reactions or 0 for irreversible reactions, and *v_ub_* is set to +inf for all reactions. In addition to searching for a flux distribution, MOMENT searches for a vector of enzyme concentrations, denoted *g* (mmol/g_DW_), such that each flux rate in *v* has a sufficiently high enzyme concentration to catalyze it. To associate flux rates with enzyme concentrations, we utilize the Boolean gene-to-reaction mapping that is included in the *E.coli* model of Feist et al. [19], as follows:

1. For a reaction *j* catalyzed by single enzyme *i*, we use the equation:
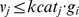

2. For a reaction *j* catalyzed by two isozymes *a* OR *b*, we use the equation:
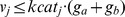

3. For a reaction *j* catalyzed by an enzyme complex consisting of gene products *a* AND *b*, we use the equation:
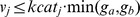

This can be formulated in a linear equation by defining an auxiliary variable *g_a&b_* that is constrained to be smaller than both *g_a_* and *g_b_*.

To account for more complex gene-to-reaction mappings, where multiple alternative enzyme complexes can catalyze a certain reaction, we applied the above rules recursively by adding auxiliary variables for AND and OR operators. For enzymes whose turnover number is unknown, we use the me median turnover number across all reactions in *E. coli* (see Results).

The enzymes solvent capacity constraint is formulated as;
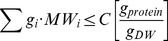
where, *MW_i_* denotes the molecular weight of protein coded by gene *i*, and *C* denotes the total weight of proteins, which was assumed to be 56% out of the *E. coli* dry weight mass [30]. Notably, the latter constraint resembles the molecular crowding constraint employed by [9], [12], [21], though here, the gene-to-reactions mapping is taken into account.

### Maximum ATP per sum of flux square

To maximize ATP yield per sum of flux square, 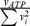, as performed by Schuetz et al. [18], requies non-convex optimization.To overcome that, we utilized the same approach suggested by Schuetz at el. [18], and solved a series of quadratic programming optimization problems of the form:
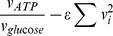
where *ε* represents a trade-off between ATP maximization and minimization of flux norm. Specifically, we iterate over 10000 values of ε between 0.5 to 1.5 to identify the optimal flux distribution that maximizes ATP yield per flux unit.

## Supporting Information

Figure S1 (A) Histogram of Pearson correlations between enzyme turnover numbers and predicted flux rates, uniquely determined by Flux Variability Analysis (FVA), under different single carbon and energy source media in blue (average Pearson correlation is 0.49; *p*-value = 0.002). The Pearson correlation between enzyme turnover numbers and the averaged flux distribution across conditions in green (Pearson correlation of 0.55; *p*-value = 0.0001) is shown to be higher than those obtained under the different media. (B) Pearson correlations between enzyme turnover numbers and predicted fluxes whose flux rate is uniquely determined by FVA, under a set of single carbon and energy source media under either aerobic versus anaerobic conditions. As shown, under most growth conditions, the correlation between enzyme turnover numbers and fluxes is higher when fluxes are predicted under aerobic conditions(paired Wilcoxon test *p*-value = 2.3e-14).
(TIF)

Figure S2 The prediction of growth rates by FBAwMC. FBAwMC predicted growth rates achieving a Pearson correlation of 0.29(p-value = 0.17) with the measured growth rates.
(TIF)

Figure S3 Metabolic flux and gene expression level predictions via MOMENT. (A) The prediction of flux rates in *E.coli* under glucose minimal media based on MOMENT. Linear regression line in red. (B) MOMENT prediction of differential gene expression levels in *E.coli* under glucose minimal media, between low and high growth rate conditions (with the high growth rate condition involving overflow metabolism). Linear regression line in red.
(TIF)

Table S1 Correlations of enzyme turnover numbers, enzyme molecular weights, gene expression levels, and combinations of the latter via a linear regression model with metabolic flux rates.
(XLSX)

Table S2 The contribution of kinetics parameters to the prediction of metabolic flux in *E. coli* under glucose minimal media and differential gene expression.
(XLSX)

Table S3 Measured and predicted growth rates.
(XLSX)

Table S4 The prediction accuracy of growth rate predictions made by MOMENT versus FBAwMC.
(XLSX)

Table S5 Metabolic flux predictions in *E. coli* under glucose minimal media.
(XLSX)

Table S6 Prediction of differential gene expression levels in *E. coli* under glucose minimal media.
(XLSX)

Table S7 Turnover numbers.
(XLSX)

Text S1 FVA formulation for MOMENT and the relation between growth rate and the total enzyame mass.
(DOC)
