# Supplementary figures and images for: Prediction of Microbial Growth Rate versus Biomass Yield by a Metabolic Network with Kinetic Parameters
Source: PLoS Comput Biol. 2012 Jul 5;8(7):e1002575. doi: 10.1371/journal.pcbi.1002575 (PMC3390398; doi:10.1371/journal.pcbi.1002575)

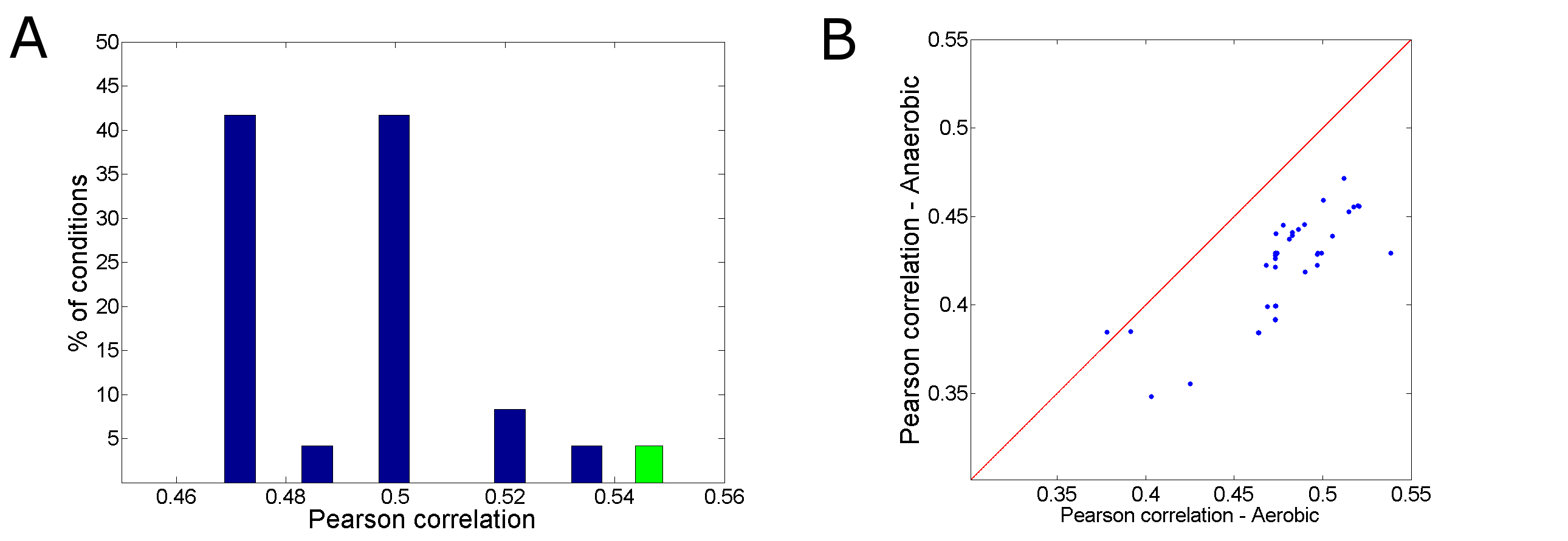

Supplement: Figure S1 — (A) Histogram of Pearson correlations between enzyme turnover numbers and predicted flux rates, uniquely determined by Flux Variability Analysis (FVA), under different single carbon and energy source media in blue (average Pearson correlation is 0.49; p-value = 0.002). The Pearson correlation between enzyme turnover numbers and the averaged flux distribution across conditions in green (Pearson correlation of 0.55; p-value = 0.0001) is shown to be higher than those obtained under the different media. (B) Pearson correlations between enzyme turnover numbers and predicted fluxes whose flux rate is uniquely determined by FVA, under a set of single carbon and energy source media under either aerobic versus anaerobic conditions. As shown, under most growth conditions, the correlation between enzyme turnover numbers and fluxes is higher when fluxes are predicted under aerobic conditions(paired Wilcoxon test p-value = 2.3e-14). (TIF) [file pcbi.1002575.s001.tif]

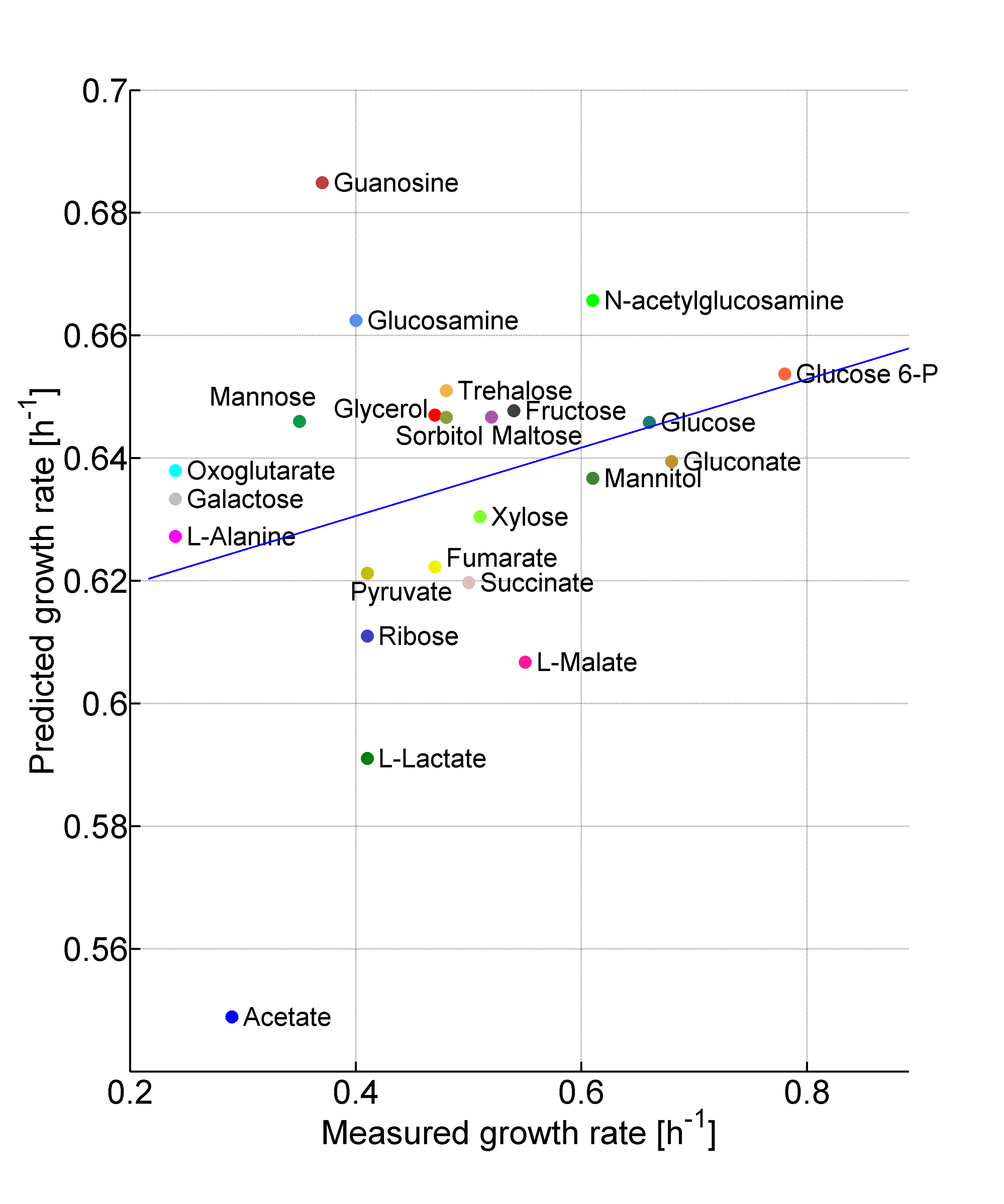

Supplement: Figure S2 — The prediction of growth rates by FBAwMC. FBAwMC predicted growth rates achieving a Pearson correlation of 0.29(p-value = 0.17) with the measured growth rates. (TIF) [file pcbi.1002575.s002.tif]

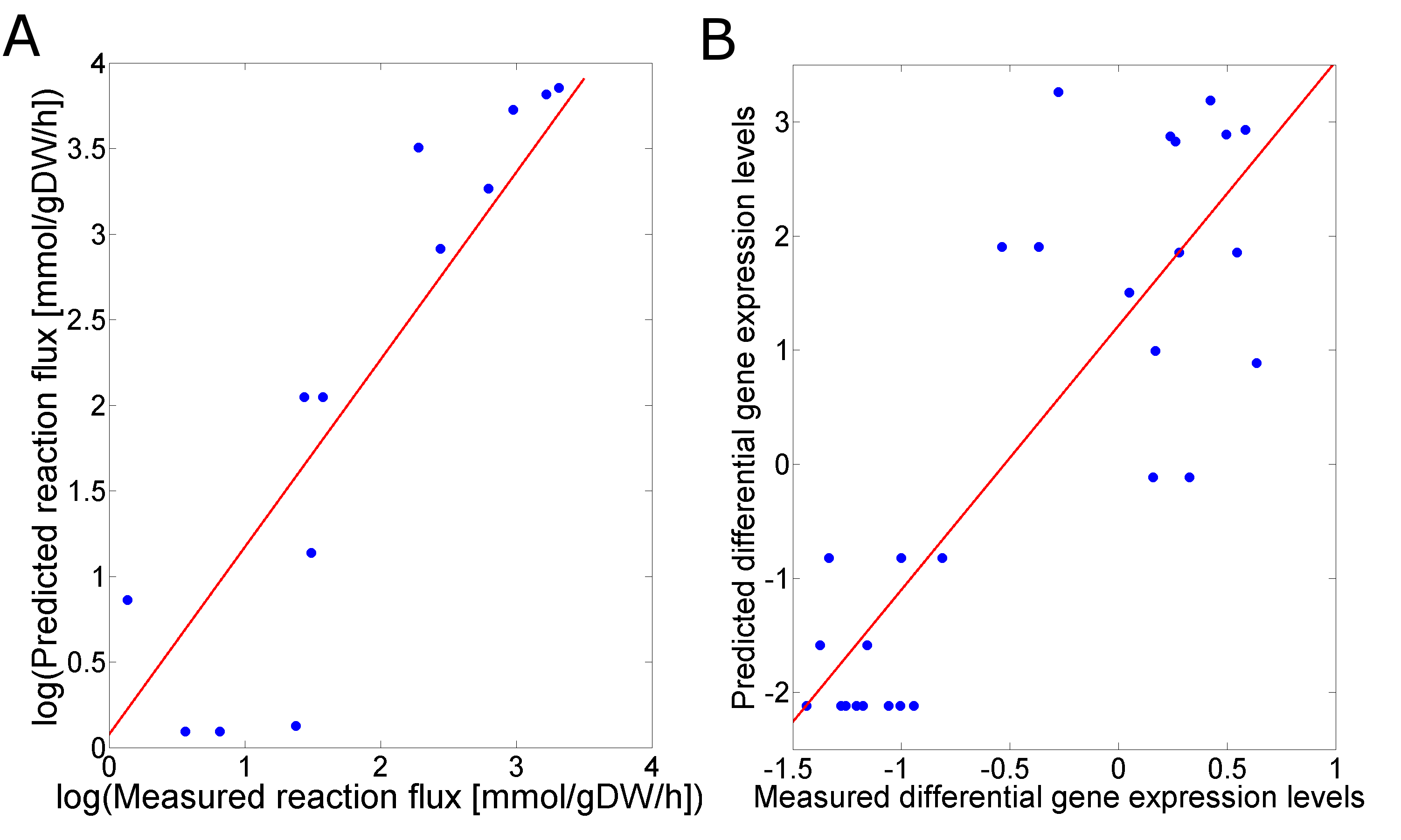

Supplement: Figure S3 — Metabolic flux and gene expression level predictions via MOMENT. (A) The prediction of flux rates in E.coli under glucose minimal media based on MOMENT. Linear regression line in red. (B) MOMENT prediction of differential gene expression levels in E.coli under glucose minimal media, between low and high growth rate conditions (with the high growth rate condition involving overflow metabolism). Linear regression line in red. (TIF) [file pcbi.1002575.s003.tif]
