# Supplementary material for: Prediction of Microbial Growth Rate versus Biomass Yield by a Metabolic Network with Kinetic Parameters
Source: PLoS Comput Biol. 2012 Jul 5;8(7):e1002575. doi: 10.1371/journal.pcbi.1002575 (PMC3390398; doi:10.1371/journal.pcbi.1002575)
Supplement: Text S1 — FVA formulation for MOMENT and the relation between growth rate and the total enzyame mass. (DOC) [file pcbi.1002575.s011.doc]

**TEXT S1**

**FVA formulation** **for MOMENT**

The vector of enzyme concentrations, *g*, is unique due to the fact that each reaction is associated with a unique gene to reaction mapping, having different turnover number and molecular weight. We confirm this by applying a variant of Flux Variability
Analysis (FVA) on MOMENT’s enzyme concentration vector, as following:

$$\max/\min g_{i}$$

$$\text{subject to }$$

$$Sv=0,$$

$$v_{lb}\leq v\leq v_{ub,}$$

$v_{j}\leq kcat_{j}\cdot g_{i}$… 🡸 genes-to-reactions constraints,

$\sum g_{i}\cdot MW_{i}\leq C\left[\frac{g_{protein}}{g_{DW}}\right]$,

$$c^{T}v=Z$$

Where *Z* is the optimal solution to the MOMENT problem:

$$\max$$

$$c^{T}v$$

$$\text{subject to }$$

$$Sv=0,$$

$$v_{lb}\leq v\leq v_{ub,}$$

$v_{j}\leq kcat_{j}\cdot g_{i}$… 🡸 genes-to-reactions constraints,

$$\sum g_{i}\cdot MW_{i}\leq C\left[\frac{g_{protein}}{g_{DW}}\right]$$

And *c* represents the biomass production’s objective function.

**The growth rate scales linearly with the threshold on the total enzyme mass**

Changing the total enzyme mass in the model scales the growth rate linearly and therefore does not change the Pearson correlation between the measured and the predicted growth rate. If we denote by *f* the flux distribution obtained by MOMENT when using a threshold *C* on the total enzyme mass, then *αf* would be an optimal MOMENT solution when using a threshold of *αC*. Notably, this results from the fact that *v_lb_* and *v_ub_* are either –inf, 0, or +inf for all reactions v in the model – as we assume that specific data on nutrient uptake and secretion rates is unavailable.
